# Constraints on Tone Sensitivity in Novel Word Learning by Monolingual and Bilingual Infants: Tone Properties Are More Influential than Tone Familiarity

**DOI:** 10.3389/fpsyg.2017.02190

**Published:** 2018-01-04

**Authors:** Denis Burnham, Leher Singh, Karen Mattock, Pei J. Woo, Marina Kalashnikova

**Affiliations:** ^1^The MARCS Institute for Brain, Behaviour and Development, Western Sydney University, Sydney, NSW, Australia; ^2^Department of Psychology, National University of Singapore, Singapore, Singapore; ^3^School of Social Sciences and Psychology, Western Sydney University, Sydney, NSW, Australia; ^4^Department of Psychology, Sunway University, Kuala Lumpur, Malaysia

**Keywords:** word learning, lexical tone, monolingual/bilingual, infant, nativenan-native

## Abstract

This study compared tone sensitivity in monolingual and bilingual infants in a novel word learning task. Tone language learning infants (Experiment 1, Mandarin monolingual; Experiment 2, Mandarin-English bilingual) were tested with Mandarin (native) or Thai (non-native) lexical tone pairs which contrasted static vs. dynamic (high vs. rising) tones or dynamic vs. dynamic (rising vs. falling) tones. Non-tone language, English-learning infants (Experiment 3) were tested on English intonational contrasts or the Mandarin or Thai tone contrasts. Monolingual Mandarin language infants were able to bind tones to novel words for the Mandarin High-Rising contrast, but not for the Mandarin Rising-Falling contrast; and they were insensitive to both the High-Rising and the Rising-Falling tone contrasts in Thai. Bilingual English-Mandarin infants were similar to the Mandarin monolinguals in that they were sensitive to the Mandarin High-Rising contrast and not to the Mandarin Rising-Falling contrast. However, unlike the Mandarin monolinguals, they were also sensitive to the High Rising contrast in Thai. Monolingual English learning infants were insensitive to all three types of contrasts (Mandarin, Thai, English), although they did respond differentially to tone-bearing vs. intonation-marked words. Findings suggest that infants' sensitivity to tones in word learning contexts depends heavily on tone properties, and that this influence is, in some cases, stronger than effects of language familiarity. Moreover, bilingual infants demonstrated greater phonological flexibility in tone interpretation.

## Introduction

The use of pitch is ubiquitous in human languages (Gussenhoven, 2004). However, the functions served by pitch variation differ markedly across languages. The majority of the world's languages spoken by the majority of the world's population (Fromkin, 1978; Yip, 2002) use pitch to differentiate the meanings of words. These languages include classic tone languages, such as Mandarin Chinese and Thai, grammatical tone languages such as Yoruba and Sesotho, as well as pitch accent languages, such as Japanese and Swedish. In all these languages, the use of pitch (as well as other cues to some extent) is applied at the syllable level to alter the meanings of words. However, pitch is also used across all the world's languages to communicate relevant information such as a speaker's emotional state, their communicative intent, and words they intend to stress (Fernald and Mazzie, 1991; Banse and Scherer, 1996; van Heuven and Haan, 2002). The multiplexing of pitch in human languages can therefore potentially introduce challenges for the young language learner. Learners of tone languages must differentiate lexical changes in pitch (i.e., lexical tone) from non-lexical changes in pitch (e.g., intonation, shifts in vocal emotion, stress, and intent), appreciating the distinct functions served by each source of pitch variation. Learners of non-tone languages must attune to the fact that their language incorporates pitch variation, but that this variation does not signal lexical contrast. Moreover, bilingual learners of both a tone and of a non-tone language, such as Mandarin Chinese and English, must learn that tone serves a different set of functions in each of their languages. As a result, they must differentiate the various functions of pitch in a language-selective manner. The focus of the current study is to determine how early word learners negotiate different types of native and non-native (lexical and non-lexical) pitch variation in relation to their language background when learning new words.

Prior research has investigated infants' sensitivity to lexical tone variation in infancy primarily via speech discrimination and novel word learning paradigms. Research in speech discrimination has focused on the basic question of whether infants of different language backgrounds (specifically, tone and non-tone language exposure) demonstrate sensitivity to lexical tone contrasts. This research complements a long tradition of research conducted with vowels and consonants that shows that infants demonstrate perceptual narrowing over the first year for many phonetic contrasts, as revealed by a selective sensitivity to vowel and consonant contrasts that feature in their native language and a reduced sensitivity to those that do not (Eimas et al., 1971; Werker and Tees, 1984; Polka and Werker, 1994; but see Best and Tyler, 2007). Studies on infant perception of lexical tones have yielded mixed findings. Firstly, some studies suggest that lexical tone undergoes a similar developmental progression to that charted for vowels and consonants; that in their first year infants raised in a tone language environment remain sensitive to lexical tone contrasts whereas those raised in a non-tone language environment demonstrate reduced sensitivity to lexical tone contrasts. Specifically, in a tone discrimination study, Mattock and Burnham (2006) investigated Thai lexical tone discrimination in Chinese (Cantonese and Mandarin) and English learning infants at 6 and 9 months of age. They found that only Chinese learning infants remain sensitive to lexical tone contrasts at 9 months and that English learning infants demonstrate a decline in sensitivity to lexical tone contrasts at 9 months. Interestingly, tone-exposed infants demonstrated sustained sensitivity to lexical tone contrasts even though the tones on which they were tested were non-native (Thai) tones. This points to broad-based early sensitivity to lexical tones in tone-exposed infants that may not be specific to the native tone inventory. In a similar study, Yeung et al. (2013) tested English, Mandarin, and Cantonese exposed infants at 4- and 9-months on Cantonese lexical tones. Like Mattock and Burnham (2006) (and repeated in Mattock et al., 2008), Yeung et al. (2013) reported a decline in discrimination of lexical tones at 9 months in English learning infants. They also reported sustained tone sensitivity in Mandarin and Cantonese infants at 4 and 9 months. However, even at 4 months Mandarin and Cantonese infants responded in different ways to one of the Cantonese tone contrasts used, which the authors interpreted as evidence for specific effects of the native tone inventory on tone perception within tone language learners. Their findings therefore point to language-selective perception of lexical tones within tone language learners.

Secondly, and in contrast to the studies described above, there is evidence opposing the emergence of language-selective sensitivity to tones in infancy. In particular, in a study of Mandarin tone perception in Dutch-exposed infants, Liu and Kager (2014) reported U-shaped development in infants' sensitivity to Mandarin lexical tones between 5 and 18 months; infants demonstrated strong tone sensitivity prior to 8 months and after 12 months. In a second study, in which they presented infants with very subtle Mandarin tone contrasts, only 5–6- and 17–18-month-old infants showed discrimination. Likewise, when presented with a different pair of Mandarin tones, Chen and Kager (2015) reported an *increase* in tone sensitivity in Dutch learning infants between 4 and 12 months. In a more recent study investigating Dutch infants' sensitivity to Limburgian tones, Ramachers et al. (2017) reported a similar increase in sensitivity to lexical tones in Dutch-exposed infants between 6 and 12 months.

Speech discrimination tasks provide clear evidence that tone-exposed infants remain sensitive to lexical tone during infancy, although it remains unclear whether they are selectively sensitive to native (vs. non-native) tone contrasts. What is less clear is whether non-tone exposed infants demonstrate a decline in sensitivity to lexical tones, with some studies demonstrating a decline (Mattock and Burnham, 2006; Mattock et al., 2008; Yeung et al., 2013) and others a temporary decline (Liu and Kager, 2014) or facilitation with increasing age (Chen and Kager, 2015; Ramachers et al., 2017). One interpretation of studies showing sustained or increased sensitivity to tones in non-tone language learners would be that these learners maintain a similar lexical interpretation of tones to their tone learning counterparts. This question can be more directly addressed by investigating how infants incorporate lexical tone variation into the process of learning new words in relation to their language background.

Past studies investigating tone sensitivity in novel word learning provide convergent evidence that both tone- *and* non-tone language learning infants demonstrate high sensitivity to lexical tones. In a study on novel word learning using a preferential looking paradigm, Singh et al. (2014) found that both bilingual infants learning English and Mandarin, and non-tone language learning infants (either monolingual in one or bilingual in two non-tone languages) incorporated lexical tones into newly learned words at 18 months. Only at 24 months did non-tone language learning infants disregard lexical tone variation when learning new words. Similarly, in a study investigating lexical tone sensitivity in novel word learning using the habituation-based Switch paradigm, Hay et al. (2015) demonstrated that English learning infants integrated Mandarin lexical tones into newly-learned words at 17 months but not at 19 months. Interestingly this period of tone sensitivity in non-tone language learning infants was extended if infants were learning *two* non-tone languages. Thus, instead of the transition from incorporating to disregarding tone in word learning occurring between 17 and 19 months (Hay et al., 2015), for bilingual non-tone learning infants this change occurs between 19 and 22 months (Estes and Hay, 2015). These findings point to differences in tone sensitivity between monolinguals and bilinguals between 19 and 22 months, even though both groups were learning non-tone languages. Further differences between monolingual and bilingual learners with respect to lexical tone sensitivity were reported by Singh et al. (2016). In this study, monolingual Mandarin learners at 12–13 months were compared with bilingual English-Mandarin learners at the same age for their sensitivity to lexical tones when learning novel words. Results revealed that bilingual English-Mandarin learners were more sensitive to lexical tones when learning words in Mandarin than their Mandarin monolingual counterparts. This was not attributed to greater tone sensitivity in bilinguals in general, as the same bilingual infants were not sensitive to Mandarin lexical tones when learning words in English. In contrast, Mandarin monolingual learners only demonstrated a similar degree of sensitivity to Mandarin tones when learning words in Mandarin 6 months later at 18 months.

Prior investigations comparing monolingual and bilingual infants on their understanding of native sound-to-meaning relations point to greater phonological flexibility in bilingual infants. As discussed above, Estes and Hay (2015) demonstrated a prolonged period of flexibility in bilingual infants' interpretation of pitch variation. Past studies investigating sensitivity to consonants have converged upon a similar conclusion: while all infants demonstrate perceptual narrowing of consonants over the first year of life, there is evidence for a postponement (i.e., delayed onset) (Garcia-Sierra et al., 2011; Ferjan Ramírez et al., 2017) as well as a protraction (i.e., delayed offset) in this process in bilingual infants (Petitto et al., 2012). Empirical reports of prolonged phonological flexibility in bilingual infants have led to conclusions that bilingualism may lead to greater phonological openness such that early learners are less tethered to the native phonological inventory (Kuhl et al., 2008). Indeed, prior studies suggest that bilingual infants continue to incorporate non-native phonological variation into newly learned words when monolingual infants no longer do so (Estes and Hay, 2015; Singh, 2017). This suggests that a prolonged course of perceptual narrowing in bilingual infants may lead to bilingual infants accepting a broader range of variation as lexically relevant when learning new words.

Findings from novel word learning studies suggest that infants from varied language backgrounds demonstrate early sensitivity to lexical tone contrasts. However, these studies have relied exclusively on sensitivity to Mandarin tones and also to a particular Mandarin tone contrast. Specifically, conclusions by Estes and Hay (2015), Singh et al. (2014), and Hay et al. (2015) were based on infants' sensitivity to a single tone contrast—the Mandarin rising/falling contrast, which is significant for the interpretation of their findings. Rising/falling pitch contours draw an important pragmatic distinction in English, Mandarin, and many other languages, specifically, the question/statement difference (Bolinger, 1958). Moreover, infants are highly sensitive to this distinction even if they are not learners of a tone language (Frota et al., 2014). This raises the possibility that tone- and non-tone language learning infants may be sensitive to the pragmatic functions of this distinction rather than to lexical tone distinctions. It remains to be seen whether these sensitivities generalise (i) to non-native tone inventories and (ii) to other Mandarin tone contrasts. That is, it is important to know (i) whether tone sensitivity in word-learning paradigms is language-specific or whether learners of tone languages possess a broad-based sensitivity to tones, and (ii) whether tone word learning is dependent on the pitch contour properties (relatively static or more dynamic) and whether such pitch characteristics of tones might override effects of nativeness.

Regarding tone familiarity, tone language infants' sensitivity to lexical tones has been consistently observed in prior studies (Mattock and Burnham, 2006; Mattock et al., 2008; Yeung et al., 2013; Liu and Kager, 2014; Chen and Kager, 2015; Ramachers et al., 2017). However, only one of these (Mattock and Burnham, 2006) suggests that tone discrimination generalizes to non-native (unfamiliar) tones. Regarding tone properties, some infant tone discrimination studies (Mattock and Burnham, 2006; Liu and Kager, 2014; Chen and Kager, 2015; Ramachers et al., 2017) show differential performance with more confusable (similar pitch direction) vs. less confusable tones, but this has yet to be investigated in tone-based word-learning studies. Moreover, the influence of bilingualism on tone sensitivity remains unclear. Although findings reported by Singh et al. (2016) point to a bilingual advantage in tone sensitivity for some Mandarin tones, it remains unknown whether this advantage extends to other tone pairs and to non-native tone contrasts.

In this study, we investigated the role of (i) tone familiarity (native vs. non-native tones), (ii) language background (monolingual/bilingual), and (iii) pitch properties of tones (static-dynamic/dynamic-dynamic) in novel word learning. Infants were tested using the Switch paradigm at 17 months given that prior studies have demonstrated effects of language background on tone sensitivity at 17–18 months using this paradigm (Estes and Hay, 2015; Hay et al., 2015). Three experiments were conducted to investigate tone-based word learning of native vs. non-native tones in 17-month-olds monolingual infants acquiring a tonal language (Mandarin, Experiment 1), bilingual infants acquiring a tonal and a non-tonal language (Mandarin-English, Experiment 2), and monolinguals infants acquiring a non-tonal language (English, Experiment 3). Tone familiarity was manipulated by varying the language of the stimuli. For Mandarin monolinguals and Mandarin-English bilinguals, native Mandarin contrasts and non-native Thai lexical tone contrasts were used. For English monolinguals, English intonational contrasts and non-native Thai and Mandarin lexical tone contrasts were used.

As different language learners use different cues to differentiate tones (see for example, Burnham and Francis, 1997; Burnham et al., 2014), and as we wished to keep tone contrasts acoustically similar across the native (Mandarin) and non-native (Thai) language stimuli, we used *a priori* bases to characterize pitch contrasts. The first was whether the tones in any particular tone contrast differed in their overall pitch movement, i.e., whether pitch was relatively static or dynamic over time and, if dynamic, then the direction of the contour was also used to characterize tones.

**Figure 1:**
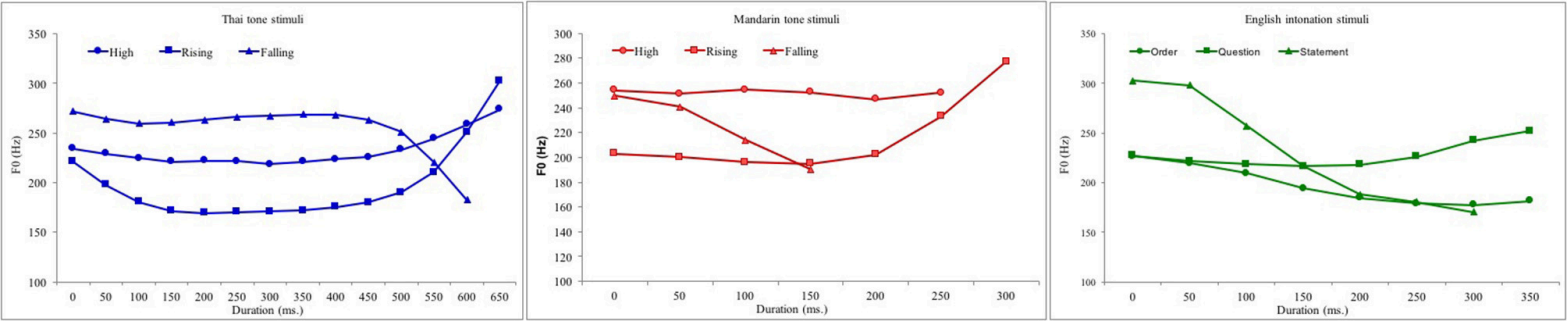
Plots of fundamental frequency (F_0_ in Hz) over time (50 ms intervals) for the Mandarin **(left)**, Thai **(central)**, and English **(right)** syllables.

Using Chao values, in which numbers are used to signal tone height at initial, (mid), and final time points, Mandarin and Thai both have High tones (Thai 45, Mandarin 55), Rising tones (Thai 315, Mandarin 35 and 214), and Falling tones (Thai 241, Mandarin 51) [with Thai also having Mid (33) and Low (21) tones, which do not match easily with Mandarin tones]. The Static-Dynamic, High vs. Rising, contrast was chosen as a contrast on which the members of the pair differed in the degree of contour—relatively static (High) and relatively dynamic (Rising)—55 vs. 35 in Mandarin; and 45 vs. 315 in Thai). The Dynamic-Dynamic, Rising vs. Falling, contrast was chosen as the other tone contrast in each language because while both are relatively dynamic, their contour direction is in the opposite direction over time in both Mandarin (35 vs. 51) and in Thai (315 vs. 241). Lexical tone is not used in English, but for comparison purposes intonation contours were used that approximate the same tone contours used in Mandarin and Thai. A Static-Dynamic pair, Order- vs. Statement-shaped syllables and a Dynamic vs. Dynamic pair, Statement- vs. Question-shaped syllables were used. These can be characterized as Mid/Falling vs. High/Falling and High-Falling vs. Mid/Rising, respectively. While these do not exactly match the High-Rising and Rising-Falling tones, use of these intonational contrasts ensured that each group heard a pitch contrast that formed a part of native language input. The intonation contours were only used for the English monolingual group to investiage if they were differentially sensitive to native English contours or non-native Mandarin or Thai contours (tones). Plots of the three Mandarin and three Thai lexical tones and the three English intonataion contours are shown in Figure 1 in the General Methods section.

### Predictions

It was predicted that bilingual and monolingual Mandarin infants would demonstrate similar levels of sensitivity to Mandarin lexical tones. Although Singh et al. (2016) demonstrated that at 12–13 months bilingual English-Mandarin learners had greater sensitivity to Mandarin tones than monolingual Mandarin learners, by 17–18 months monolingual Mandarin learners showed the same level of sensitivity to Mandarin lexical tones. Given that infants were tested at 17 months here, it was predicted that monolingual Mandarin and bilingual English-Mandarin learners would have similar levels of ability with Mandarin tones. However, it was also predicted that bilingual infants may demonstrate additional sensitivity to non-native tone contrasts (Thai) in view of past research attesting greater phonological flexibility in bilingual infants in segmental (Garcia-Sierra et al., 2011; Petitto et al., 2012; Ferjan Ramírez et al., 2017; Singh, 2017) and suprasegmental perception (Estes and Hay, 2015). Effects of tone contrast due to differences in pitch properties of tones were also predicted on account of prior studies demonstrating contrast-specific effects on the order of acquisition of individual Mandarin tones (Wong et al., 2005; Wong, 2012a,b, 2013). Finally, differences in effects of nativeness and pitch properties of tone pairs across monolingual and bilingual groups will be explored.

## General methods

Methods common to the three experiments are set out ahead of specific methods for each.

### Materials and apparatus

The stimuli for each of the three experiments in each of the four conditions (native/non-native × Static-Static/Static/Dynamic) are set out in Table 1, and their fundamental frequency contours are shown in Figure 1.

**Table 1:** Language and Tone Contrast Familiarity and Tone Contrast Properties used in the four conditions of the habituation then switch task in Experiments 1, 2, and 3.

**Expt: Infants**	**Condition**			**Stimuli in each language**
	**Familiarity**	**Contrast type**	**Contrast pitch**	
Expt. 1: Monolingual Mandarin	Native	Static-Dynamic	High-Rising	Mandarin T1 [55] vs. T2 [35]
	Native	Dynamic-Dynamic	Rising-Falling	Mandarin T2 [35] vs. T4 [51]
	Non-Native	Static-Dynamic	High-Rising	Thai High [45] vs. Rising [315]
	Non-Native	Dynamic-Dynamic	Rising-Falling	Thai Rising [315] vs. Falling [241]
Expt. 2: Bilingual Mandarin-English	Native	Static-Dynamic	High-Rising	Mandarin T1 [55] vs. T2 [35]
	Native	Dynamic-Dynamic	Rising-Falling	Mandarin T2 [35] vs. T4 [51]
	Non-Native	Static-Dynamic	High-Rising	Thai High [45] vs. Rising [315]
	Non-Native	Dynamic-Dynamic	Rising-Falling	Thai Rising [315] vs. Falling [241]
Expt. 3: Monolingual English	Native	Static-Dynamic	Mid/Falling-High/Falling	Order vs. Statement
	Native	Dynamic-Dynamic	High-Falling vs. Mid/Rising	Statement vs. Question
	Non-Native	Static-Dynamic	High-Rising	*Counterbalanced between Ss:* Mandarin T1 [55] vs. T2 [35] Thai High [45] vs. Rising [315]
	Non-Nat	Dynamic-Dynamic	Rising-Falling	*Counterbalanced between Ss:* Mandarin T2 [35] vs. T4 [51] Thai Rising [315] vs. Falling [241]

A native female speaker of Malaysian Mandarin was audio-recorded producing the syllable /kha/ (Pinyin “ka”) with the Mandarin tones T1 [55] and T2 [35] and T4 [51], in order to create a Static-Dynamic contrast, [55-35] and a Dynamic-Dynamic contrast, [35-51]. Only one of these syllables is a word in Mandarin, but it is of low frequency and is certainly not a word that would be high frequency in speech addressed to infants—[kha55] is a homophone (a) 咖 the first noun in the compound word meaning *coffee* (frequency = 4,366, percentile = 76.0, where 1 is the lowest and 100 percentile the highest possible frequency) or (b) 喀 *onomatopoeic* of the coughing sound (frequency = 3,830, percentile = 75.1). The other two used here, [kha35] and [kha51], are not words in Mandarin (Da, 2015).

A native female speaker of Thai was audio-recorded producing the syllable /khaa/ with the Thai tones High [45], Rising [315], and Falling [241], in order to create a Static-Dynamic contrast, [45-315] and a Dynamic-Dynamic contrast, [315-241]. All three of these are words in Thai—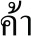 [khaa45] means *to trade*; 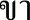 /khaa315/ means *leg*; and [khaa241] is a homophone, meaning (a) 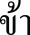 *I, me* (this is antiquated) or (b) 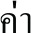 *value*; or (c) 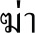 *to kill*. These are all relatively low frequency but frequency is of no concern for the Thai stimuli as they simply served as non-native stimuli for the Mandarin background and English background infants. No Thai infants were tested.

A female native English speaker was recorded producing the syllable /ka/ with the following intonation contours: statement, order, and question.

For each set of language stimuli, the syllables were extracted from the recording and concatenated into 20 s strings with an inter-stimulus interval (ISI) of 500 ms. The visual stimuli consisted of video recordings of two colorful novel objects (a molecule and a crown) moving slowly along the horizontal axis in the center of the screen. Additionally, a video of a moving toy (spinning water-wheel) and an audio recording of the novel word /pok/ produced by a female speaker were used in the pre- and post-test phases of the task.

Stimuli were presented using Habit X1.0 software (Cohen et al., 2004) on a computer screen with the audio presented through loudspeakers located behind the screen. Infants sat on their caregiver's lap ~60 cm away from the screen. Caregivers listened to masking sounds through headphones. The experimenter observed the infant through a CCTV camera in an adjacent room and controlled the presentation of the stimuli.

### Procedure

**Figure 2:**
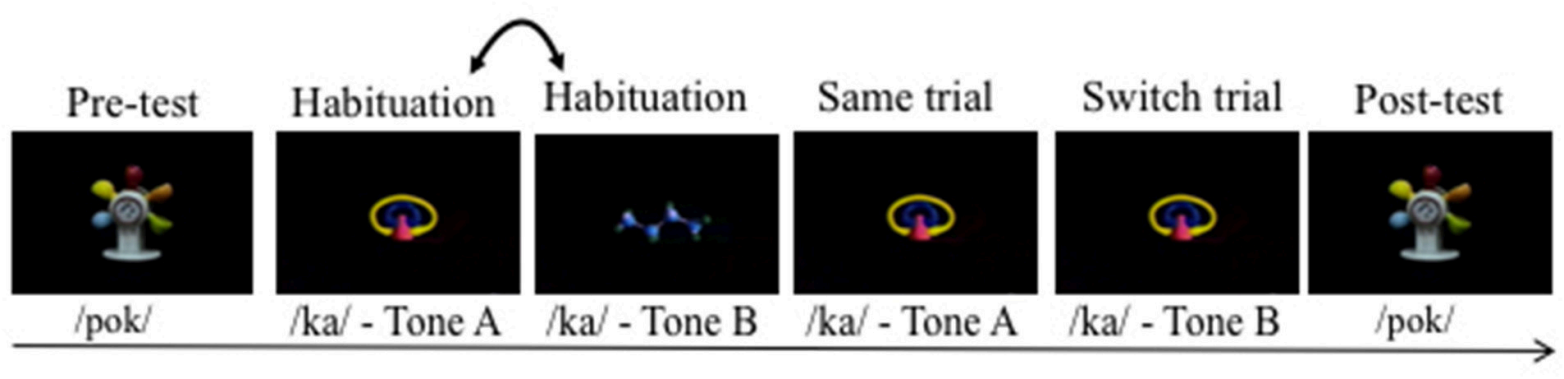
Graphical representation of the Switch task. The doubled-headed arrow indicates that /ka/-Tone A and /ka/-Tone B were presented in alternation in habituation, until the habituation criterion was reached.

Each infant completed one of the between-subjects conditions of the task: native Static-Dynamic, native Dynamic-Dynamic, non-native Static-Dynamic, or non-native Dynamic-Dynamic. At the start of the task, infants were presented with the attention getter, a flashing red light on the screen accompanied by a beeping sound. Once they had fixated the screen, the experimental task commenced. First, infants completed an habituation phase in which they saw each object (molecule or crown) paired with a different sound stimulus (e.g., crown + /ka/ Tone A, molecule + /ka/ Tone B, with the nature of Tone A and Tone B depending on the experiment). The habituation phase proceeded until infants reached the habituation criterion (decrease of 50% or more in looking time in two consecutive trials in comparison to the mean looking duration over the first three habituation trials) or after reaching the maximum of 24 habituation trials. After that, infants completed two test trials. One was a Same trial, in which the infants saw one of the object-sound pairings from the habituation phase (e.g., crown + /ka/ Tone A). The other was a Switch trial where infants saw the same object but paired with the sound that corresponded to the other object in the habituation phase (e.g., crown + /ka/ Tone B). Infants also completed a pre- and post-test trial at the start and end of each session (Figure 2). The pairings between the visual and auditory stimuli, the objects chosen for the test phase, and the order of Same and Switch trial presentation were all counterbalanced between participants.

## Experiment 1: monolingual mandarin infants

In Experiment 1, four groups of Monolingual Mandarin environment infants were tested with native Mandarin tone contrasts (High-Rising, T1 [55] vs. T2 [35]; Rising-Falling, T2 [35] vs. T4 [51]) and non-native contrasts (High-Rising, Thai [45] vs. [315]; Rising-Falling, Thai [315] vs. [241]).

### Participants

Thirty-three 17-month-old infants (16 female; *M* age = 523.06 days [17.26 months], *SD* = 13.07) participated. One additional infant participated but was excluded from final analyses due to experimenter error. Infants were randomly assigned to one of four groups: Native Rising-Falling (*n* = 9), Native High-Rising (*n* = 8), Non-native Rising-Falling (*n* = 8), Non-native High-Rising (*n* = 8). Parents were asked to complete a brief questionnaire about their infants' language environment and exposure. All infants were acquiring Mandarin as their first language and had no more than 10% exposure to any additional language (*M* = 6.08%, *SD* = 3.3) as reported by their primary caregiver. Twenty-nine infants were growing up in Singapore and four infants were growing up in Malaysia. All infants were typically-developing and were not at risk for sensory or developmental disorders.

### Results

Given that infant looking time data were not normally distributed, all raw looking time scores were subject to a log transformation, so that the data could be analyzed using Analyses of Variance.

First, infants' performance in the habituation phase and the pre- and post-test trials were compared across the four tests groups (Native High-Rising, Native Rising-Falling, Non-native High-Rising, Non-native Rising-Falling) (see Table 2, Experiment 1). Total looking duration, *F*_(3, 29)_ = 0.784, *p* = 0.513, η^2^ = 0.075, and the number of habituation trials, *F*_(3, 29)_ = 0.622, *p* = 0.607, η^2^ = 0.060, did not differ across groups. Similarly, looking duration did not differ between pre- and post-test trials, *F*_(1, 29)_ = 2.156, *p* = 0.153, η^2^ = 0.069, and there was no effect of group, *F*_(3, 29)_ = 0.722, *p* = 0.547, η^2^ = 0.069, and no significant pre-/post-trial × group interaction, *F*_(3, 29)_ = 0.943, *p* = 0.433, η^2^ = 0.089. Thus, there was no systematic bias in attention between the groups, and within groups there was no general fatigue over time—attention did not diminish between pre- and post-test trials.

**Table 2:** Mean (*SD*) habituation duration, number of habituation trials, pre- and post-test fixations in the four conditions of Experiments 1, 2, and 3.

**Experiment**	**Condition**	**Habituation duration^a^**	**Habituation (*N* trials)**	**Pre-test^a^**	**Post-test^a^**
Monolingual Mandarin (Expt. 1)	Native Static-Dynamic	1.99 (0.25)	9.88 (5.30)	1.28 (0.04)	1.16 (0.17)
	Native Dynamic-Dynamic	2.08 (0.23)	13.56 (6.25)	1.31 (0.01)	1.22 (0.12)
	Non-Native Static-Dynamic	2.17 (0.24)	12.88 (7.04)	1.21 (0.22)	1.23 (0.14)
	Non-Native Dynamic-Dynamic	2.07 (0.21)	10.88 (6.38)	1.28 (0.05)	1.25 (0.15)
Bilingual Mandarin-English (Expt. 2)	Native Static-Dynamic	2.09 (0.22)	14.5 (6.87)	1.22 (0.12)	1.19 (0.17)
	Native Dynamic-Dynamic	2.05 (0.25)	12.13 (6.22)	1.27 (0.07)	1.19 (0.18)
	Non-Native Static-Dynamic	2.08 (0.37)	13.13 (7.29)	1.21 (0.24)	1.13 (0.34)
	Non-Native Dynamic-Dynamic	1.96 (0.28)	9.87 (6.18)	1.26 (0.12)	1.14 (0.15)
Monolingual English (Expt. 3)	Native Static-Dynamic	1.93 (0.17)	11.75 (7.11)	1.25 (0.09)	1.23 (0.18)
	Native Dynamic-Dynamic	1.84 (0.24)	8.5 (4.44)	1.09 (0.20)	1.07 (0.22)
	Non-Native Static-Dynamic	1.86 (0.30)	8 (3.89)	1.14 (0.29)	1.19 (0.15)
	Non-Native Dynamic-Dynamic	1.95 (0.33)	11.14 (6.62)	1.08 (0.28)	1.25 (0.09)

a *Log-transformed looking duration (seconds)*.

**Figure 3:**
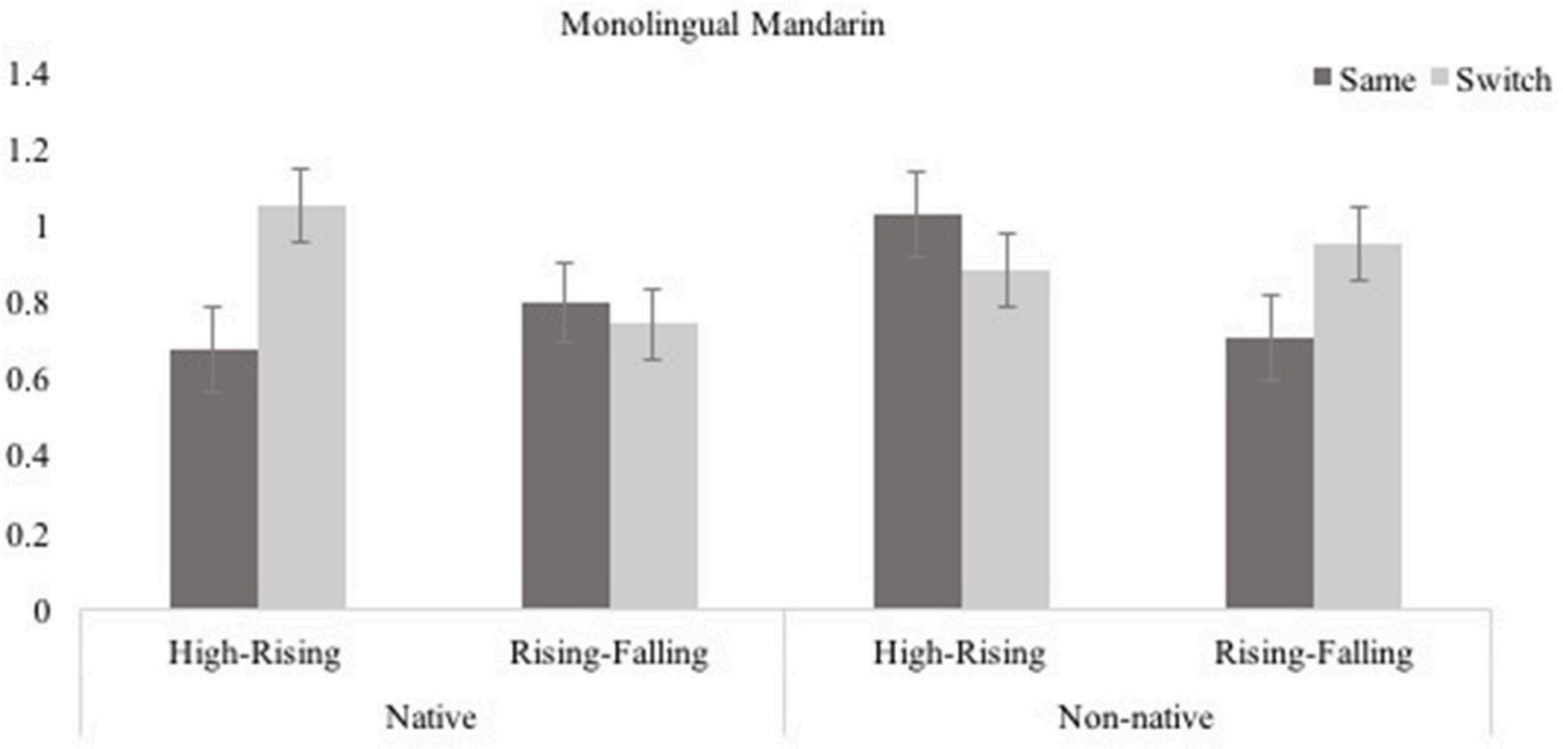
Monolingual Mandarin infants' performance in the four conditions of the Switch task (Experiment 1; error bars show SEM).

Looking times in test trials for Native/Non-native, High-Rising/Rising-Falling, and Same/Switch trials are shown in Figure 3. To assess infants' performance in these test trials, looking durations for the Same and Switch trials across the native vs. non-native and the stimulus type conditions were compared. A 2 (Native, Non-native) × 2 (High-Rising, Rising-Falling) × 2 (Same, Switch) ANOVA showed no main effect of Same/Switch, *F*_(1, 29)_ = 2.212, *p* = 0.148, η^2^ = 0.071, Native/Non-native, *F*_(1, 29)_ = 1.006, *p* = 0.324, η^2^ = 0.034, Tone Type, *F*_(1, 29)_ = 2.177, *p* = 0.151, η^2^ = 0.070, and no Same/Switch × Native/Non-native, *F*_(1, 29)_ = 0.622, *p* = 0.437, η^2^ = 0.021, Same/Switch × Tone Type, *F*_(1, 29)_ = 0.022, *p* = 0.882, η^2^ = 0.001, or Native/Non-native by Tone Type interactions, *F*_(1, 29)_ = 0.006, *p* = 0.805, η^2^ = 0.002. However, there was a significant three-way Same/Switch × Native/Non-native × tone type interaction, *F*_(1, 29)_ = 8.594, *p* = 0.007, η^2^ = 0.229 (see Figure 3).

To investigate the source of the interaction, infants' performance was analyzed separately for the static-dynamic (High-Rising) and dynamic-dynamic (Rising-Falling) conditions. In the High-Rising condition, there was no main effect of Same/Switch, *F*_(1, 14)_ = 2.094, *p* = 0.170, η^2^ = 0.130, or Native/Non-native, *F*_(1, 14)_ = 0.808, *p* = 0.384, η^2^ = 0.055, but these two factors did interact, *F*_(1, 14)_ = 10.823, *p* = 0.005, η^2^ = 0.436. Infants looked significantly longer in response to the Switch than the Same trials in the native (Same *M* = 0.671, *SE* = 0.121; Switch *M* = 1.049, *SE* = 0.083), *t*_(7)_ = −2.923, *p* = 0.022, *d* = 1.283, but not the non-native condition (Same *M* = 1.028, *SE* = 0.067; Switch *M* = 0.881, *SE* = 0.092), *t*_(7)_ = 1.572, *p* = 0.160, *d* = 0.644. On the other hand, in the Rising-Falling condition there were no main effects of Same/Switch, *F*_(1, 15)_ = 0.681, *p* = 0.422, η^2^ = 0.043 (Same *M* = 0.796, *SE* = 0.116; Switch *M* = 0.740, *SE* = 0.098), or Native/Non-native, *F*_(1, 15)_ = 0.278, *p* = 0.606, n = 0.018 (Same *M* = 0.702, *SE* = 0.123; Switch *M* = 0.947, *SE* = 0.104), and also no significant interaction, *F*_(1, 15)_ = 1.746, *p* = 0.206, η^2^ = 0.104. Thus, when learning tone-bearing words, monolingual Mandarin infants were sensitive to native but not to non-native tone contrasts in this task. However, this was only the case for the static-dynamic (High-Rising) tone pairs—they did not look significantly longer to the Switch trial for the native dynamic-dynamic (Rising-Falling) tone pair.

When learning novel words, monolingual Mandarin infants were sensitive to static vs. dynamic (High-Rising) native tones but not to dynamic-dynamic (Rising-Falling) native tones. They were not sensitive to either type of non-native tone contrast (static vs. dynamic or dynamic-dynamic tone pairs).

## Experiment 2: bilingual Mandarin-English infants

In Experiment 2, four groups of bilingual Mandarin-English environment infants were tested with the same contrasts as in Experiment 1, native tone contrasts (High-Rising, Mandarin T1 [55] vs. T2 [35]; Rising-Falling, Mandarin T2 [35] vs. T4 [51]) and non-native contrasts (High-Rising, Thai High [45] vs. Rising [315]; Rising-Falling, Thai Rising [315] vs. Falling [241]).

### Participants

Thirty-two 17-month-old infants (16 female; *M*age = 524.72 days [17.25 months], *SD* = 18.02) were included in the study. An additional five infants participated but were excluded due to failure to comply with the language selection criteria. Twenty-two infants were being raised in Singapore and ten infants were being raised in Malaysia. Infants were randomly assigned to four groups according to two between-subjects experimental conditions, native vs. non-native and High-Rising vs. Rising-Falling (Native High-Rising *n* = 8, Native Rising-Falling *n* = 8, Non-native High-Rising *n* = 8, Non-native Rising-Falling *n* = 8).

All infants were typically-developing and were not at risk for sensory or developmental disorders. Parents were asked to complete a questionnaire about their infants' language environment and exposure. Infants' weekly language exposure ranged from 26 to 72% (*M* = 51.48, *SD* = 13.69) for Mandarin and from 25 to 68% for English (*M* = 45.9, *SD* = 13.97). Sixteen children were reported to have some exposure to a third language, but this exposure was <10% (*M* = 5.6%, *SD* = 3.5%). Analysis revealed that degree of language exposure had no effect on the results^1^.

### Results

Performance in the habituation phase and pre- and post-test trials of the four between-subjects conditions revealed that infants' looking duration, *F*_(3, 28)_ = 0.339, *p* = 0.798, η^2^ = 0.035, and number of habituation trials, *F*_(3, 28)_ = 0.685, *p* = 0.569, η^2^ = 0.068, did not differ across groups. Similarly looking duration to the pre- and post-test trials did not differ, *F*_(1, 28)_ = 2.332, *p* = 0.138, η^2^ = 0.077, across groups, *F*_(3, 28)_ = 0.332, *p* = 0.803, η^2^ = 0.034, and there was no significant trial × group interaction, *F*_(3, 28)_ = 0.134, *p* = 0.939, η^2^ = 0.014. Thus, there was no systematic bias in attention between the groups, and within groups there was no general fatigue over time—attention did not diminish between pre- and post-test trials.

**Figure 4:**
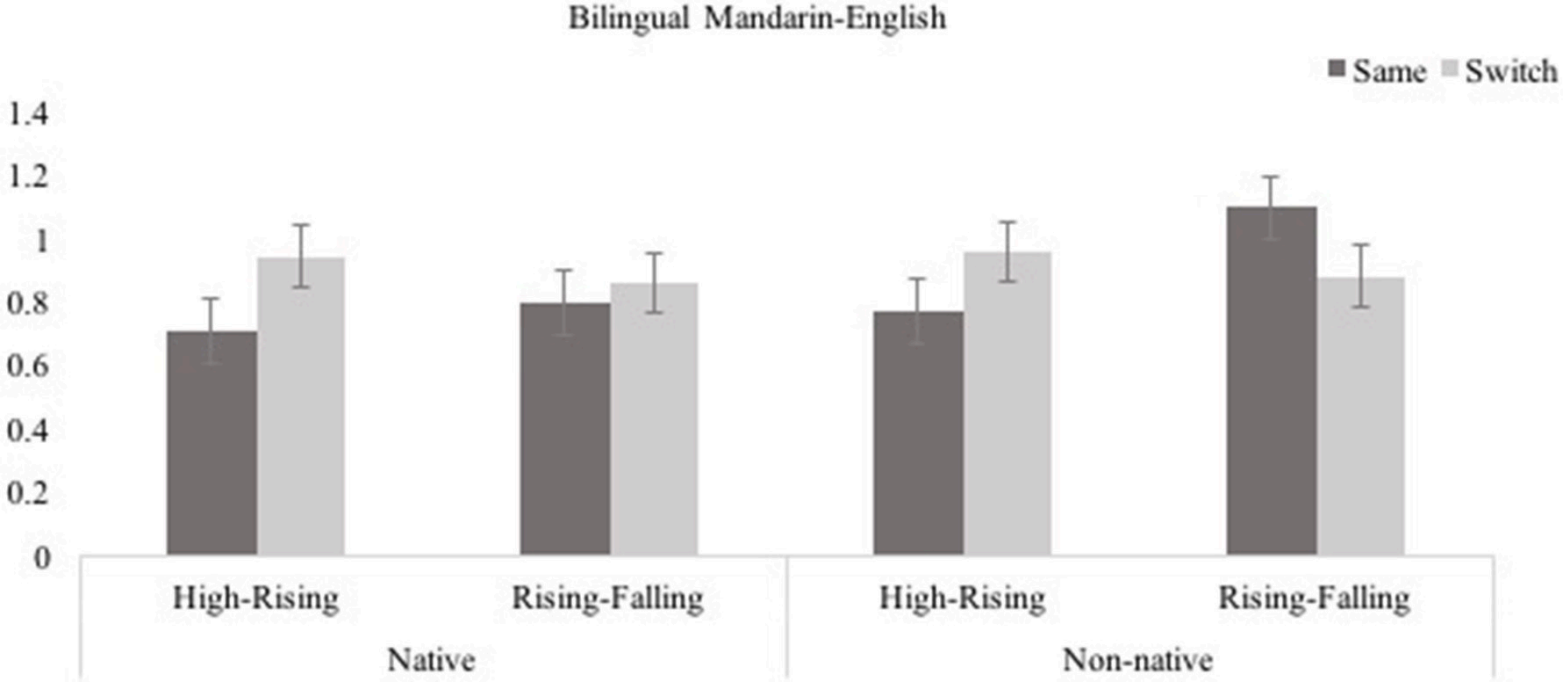
Bilingual Mandarin-English infants' performance in the four conditions of the switch task (Experiment 2; error bars show SEM).

Log transformed looking times in test trials for Native/Non-native, High-Rising/ Rising-Falling, and Same/Switch trials are shown in Figure 4. A 2 (Native, Non-native) × 2 (High-Rising, Rising-Falling) × 2 (Same, Switch) ANOVA was conducted to assess infants' performance in the test phase. There were no main effects of Same/Switch, *F*_(1, 28)_ = 1.340, *p* = 0.257, η^2^ = 0.046, Native/Non-native, *F*_(1, 28)_ = 1.553, *p* = 0.223, η^2^ = 0.053, or Tone Type, *F*_(1, 28)_ = 0.650, *p* = 0.427, η^2^ = 0.023. However, contrary to the Mandarin monolingual group, there was a significant Same/Switch × Tone Type interaction, *F*_(1, 28)_ = 6.273, *p* = 0.018, η^2^ = 0.183. All other two- and three way interactions were not significant, (Same/Switch × Native/Non-native, *F*_(1, 28)_ = 2.003, *p* = 0.168, η^2^ = 0.067, Native/Non-native × Tone Type, *F*_(1, 28)_ = 0.599, *p* = 0.445, η^2^ = 0.021, and Same/Switch × Native/Non-native × Tone Type, *F*_(1, 28)_ = 0.968, *p* = 0.334, η^2^ = 0.033).

The source of this Same/Switch × Tone Type interaction was investigated by assessing infants' performance separately in the High-Rising and Rising-Falling conditions. In the High-Rising condition, infants produced significantly longer looks in the Switch (*M* = 0.948, *SE* = 0.077) than in the Same trials (*M* = 0.736, *SE* = 0.078), *F*_(1, 14)_ = 5.004, *p* = 0.042, η^2^ = 0.263. This was the case for both native and non-native conditions, as there were no significant effects of Native/Non-native, *F*_(1, 14)_ = 0.094, *p* = 0.764, η^2^ = 0.007, nor was there a Same/Switch × Native/Non-native interaction, *F*_(1, 14)_ = 0.069, *p* = 0.796, η^2^ = 0.005. Infants looked longer in the Switch trials when they were presented with a High-Rising tone contrast, either the native Mandarin T1 [55] vs. T2 [35], or the non-native Thai High [45] vs. Rising [315] contrast. For the Rising-Falling tone types, however, there were no significant differences in infants' looking duration in the Switch (*M* = 0.567, *SE* = 0.057) and Same (*M* = 0.945, *SE* = 0.064) trials, *F*_(1, 14)_ = 1.374, *p* = 0.261, η^2^ = 0.089, and there was no Native/Non-native effect, *F*_(1, 14)_ = 2.519, *p* = 0.135, η^2^ = 0.153, and no Same/Switch × Native/Non-native interaction, *F*_(1, 14)_ = 4.631, *p* = 0.056, η^2^ = 0.238.

As for the Mandarin monolingual infants, Mandarin-English bilingual infants were not sensitive to Rising and Falling tones in one of their native languages, Mandarin, nor in a non-native tone language, Thai. However, similar to Mandarin monolingual infants they were sensitive to High and Rising tones in their own tone language, Mandarin, but unlike monolingual Mandarin infants, bilinguals *were* sensitive to non-native High and Rising tones in Thai.

## Experiment 3: monolingual English infants

In Experiment 1, Monolingual Mandarin language infants learned words on the basis of a native high-rising but not a rising-falling contrast. In Experiment 2, Bilingual Mandarin language infants learned words on the basis of a native high-rising but not a rising-falling contrast, and also on the basis of a non-native high-rising but not a rising-falling contrast. It could be that, over and above any advantage for bilingual over monolingual infants' perception of non-native tone contrasts, the high-rising contrast is particularly salient independent of tone language experience. To test this we added a third experiment in which non-tone, English, language experience infants were tested. For this group, the high rising tone is also native in that it conveys a question form, but it is non-lexical. In this sense, testing sensitivity to a high-rising contrast in addition to a rising-falling contrast serves to qualify our interpretation of the findings of Experiments 1 and 2. Specifically, if Monolingual English learning infants cannot learn words based on the high-rising contrast, then we presume selective sensitivity to this contrast in tone language learners is not stimulus driven, but is guided by phonological knowledge. In this group, we also took the opportunity to investigate sensitivity to native English intonational contrasts. The purpose of this was to address two additional questions which could not be answered by tone language learners. First, we sought to investigate whether infants only bind pitch to word meanings if their language binds pitch to word meanings, or whether they demonstrate a general sensitivity to contrastive pitch movements when learning new words even if their language does not lexicalize pitch. Prior studies (e.g., Singh et al., 2014; Hay et al., 2015) have demonstrated that English monolingual learners do bind Mandarin tones to word meaning; however, these studies were both based on sensitivity to a single rising-falling contrast. We have yet to learn whether these sensitivities are present in equal measure for other lexical tone contrasts and moreover, for native intonational contrasts. A second question derives from the fact that some pitch movements in English intonational systems—such as the question/statement contrast—correspond in pitch direction to lexical tone contrasts. Contrasting sensitivity to similar lexical and intonational contrasts in English monolingual infants may reveal whether non-tone language learning infants demonstrate a selective sensitivity to native phonogical variation in pitch or whether they maintain a generalized sensitivity to isomorphic pitch contours, native or not.

In Experiment 3, four groups of Monolingual English environment infants were tested with tone (non-native) lexical tone contrasts (High-Rising—Mandarin T1 [55] vs. T2 [35], or Thai [45] vs. [315], counterbalanced between infants; Rising-Falling, Mandarin T2 [35] vs. T4 [51], or Thai [315] vs. [241], counterbalanced between infants) (see Table 1). The native condition consisted of contrasts of English intonation: English Order vs. Statement (Mid/Falling-High/Falling), and Statement vs. Question (High-Falling vs. Mid/Rising).

### Participants

Thirty-one 17-month-old infants (22 female; *M*age = 532.39 days [17.5 months], *SD* = 12.75) were included in this experiment. An additional six infants participated but were excluded due to fussiness and failure to complete the experiment. Infants were randomly assigned to the four groups: native Order vs Statement (*n* = 8), native Statement vs. Question (*n* = 8), non-native High vs. Rising (*n* = 8, 4 tested on Mandarin and 4 on Thai tones), non-native Rising vs. Falling (*n* = 7, 4 tested on Mandarin and 3 on Thai tones). Using a parental questionnaire about infants' language environment and exposure, it was confirmed that all infants were acquiring English as their first language and had no exposure to any additional language. Twenty-nine children were growing up in the United Kingdom, and two infants were growing up in Australia. All infants were typically-developing and were not at risk for sensory or developmental disorders.

### Results

Habituation trial data are presented in Table 2 (Experiment 3). Comparison of infants' performance in the pre- and post-test and habituation phases across the four groups revealed no between-group differences in total looking duration, *F*_(3, 27)_ = 0.313, *p* = 0.816, η^2^ = 0.034, or the number of habituation trials, *F*_(3, 27)_ = 0.863, *p* = 0.472, η^2^ = 0.087. Similarly, there was no difference in looking duration in the pre- and post-test trials, *F*_(1, 27)_ = 1.023, *p* = 0.321, η^2^ = 0.037, and there was no effect of group, *F*_(3, 27)_ = 1.304, *p* = 0.293, η^2^ = 0.127, and no significant pre-/post-trial × group interaction, *F*_(3, 27)_ = 0.998, *p* = 0.409, η^2^ = 0.100. Thus, there was no systematic bias in attention between the groups, and within groups there was no general fatigue over time—attention did not diminish between pre- and post-test trials.

**Figure 5:**
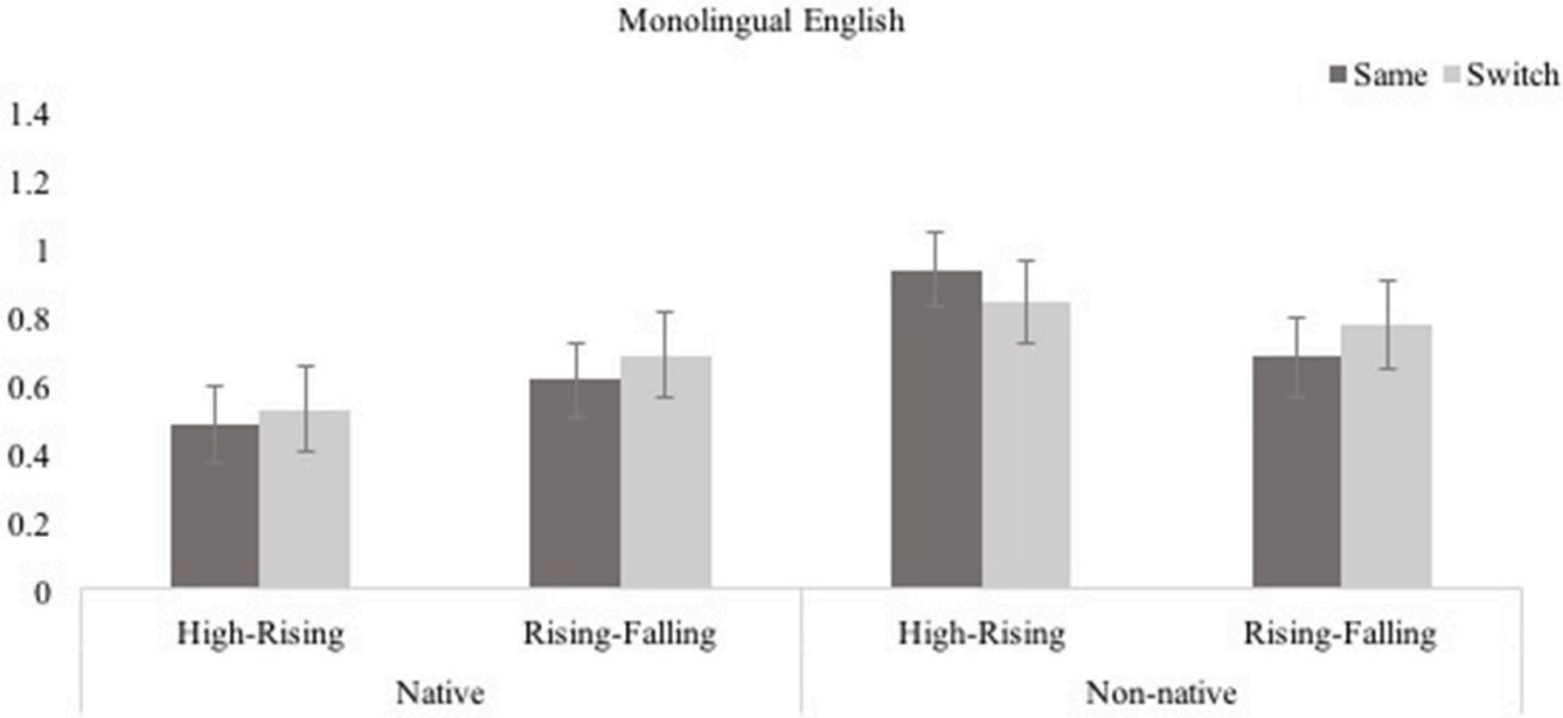
Monolingual English infants' performance in the four conditions of the switch task (Experiment 3; error bars show SEM).

Log transformed looking times in test trials for Native/Non-native, Tone Type, and Same/Switch trials are shown in Figure 5. To compare infants' performance in the test phase, looking duration for the Same and Switch trials across the native vs. non-native and the tone type conditions, a 2 (Native, Non-Native) × 2 (Static-Dynamic, Dynamic-Dynamic) × 2 (Same, Switch) ANOVA was conducted. This yielded no main effects of Same/Switch, *F*_(1, 27)_ = 0.209, *p* = 0.651, η^2^ = 0.008, and Tone Type, *F*_(1, 27)_ = 1.887, *p* = 0.181,η^2^ = 0.065. However, the main effect of Native/Non-native was significant, *F*_(1, 27)_ = 5.359, *p* = 0.028, η^2^ = 0.166. Monolingual English infants who were presented with non-native Mandarin and Thai lexical tones (*M* = 0.811, *SE* = 072) produced significantly longer looks than infants presented with native intonation contours (*M* = 0.579, *SE* = 0.010). Importantly, there were no interactions of Same/Switch × Native/Non-native, *F*_(1, 27)_ = 0.191, *p* = 0.665, η^2^ = 0.007, Same/Switch × Tone Type, *F*_(1, 27)_ = 0.718, *p* = 0.404, η^2^ = 0.026, Native/Non-native × Tone Type, *F*_(1, 27)_ = 2.366, *p* = 0.136, η^2^ = 0.081, or of Same/Switch × Native/Non-native × Tone Type, *F*_(1, 27)_ = 0.389, *p* = 0.538, η^2^ = 0.014. Infants' looking duration did not differ significantly in response to the Switch and Same trials in the native or the non-native conditions involving either the Static-Dynamic or the Dynamic-Dynamic contrasts.

Monolingual English infants were not sensitive to native intonational contrasts (Order vs.Statement, or Statement vs. Question) nor to non-native lexical tone contrasts (High vs. Rising or Rising vs. Falling) when learning novel words. While not making these fine distintions between pitch contours, they did attend to unfamiliar non-native lexical tones to a greater extent than to familiar intonation patterns.

## Discussion

The results of the three experiments are summarised in Table 3. As can be seen, each group of learners interpreted pitch movements in distinct ways. The results for each of the three groups are summarized below.

**Table 3:** Summary of word learning results in the Three Experiments (✓ = significant word learning, ✗ = no significant word learning).

**Participants**	**Native**		**Non-Native**	
	High-Rising	Rising-Falling	High-Rising	Rising-Falling
	T1-T2 (55 vs. 35)	T2-T4 (35 vs. 51)	H-R (45 vs. 315)	R-F (315 vs. 241)
Monolingual Mandarin	✓	✗	✗	✗
Bilingual Mandarin/English	✓	✗	✓	✗
	Order vs. Statement	Statement vs. Question	Mandarin (55-35)	Mandarin (35 vs. 51)
			*or* Thai (45 vs. 315)	*or* Thai (315 vs. 241)
Monolingual English	✗	✗	✗	✗
	• No discrimination of native intonations or non-native tones			
	• But greater attention to non-native tone than to native intonation			

### Monolingual Mandarin learning infants

Monolingual Mandarin learning infants only contrasted words using the Mandarin High-Rising contrast. They did not contrast words using a Mandarin Rising-Falling contrast. They also did not contrast words using Thai contrasts with similar pitch properties to Mandarin tones.

### Bilingual Mandarin-English learning infants

Bilingual Mandarin-English learning infants, like Mandarin monolinguals, demonstrated sensitivity to the Mandarin High-Rising contrast, but not to the Mandarin Rising-Falling contrast. However, unlike Mandarin monolingual learners, their sensitivity to a native Mandarin High-Rising contrast extended to the non-native Thai High-Rising tone contrast.

### Monolingual English learning infants

Monolingual English learning infants, in contrast to Mandarin-exposed infants, (both monolingual and bilingual) did not contrast words by any type of pitch contrast included in the experiment (Mandarin contrasts, Thai contrasts, intonational contrasts). Nevertheless, they were senstive to pitch in that they attended to lexical tone-bearing words to a greater extent than intonationally marked words.

These findings suggest that participants' language background as well as the pitch properties of individual pitch/tone pairs influenced pitch sensitivity in novel word learning. Below, we discuss the results of each group in turn.

### Mandarin monolinguals

Findings from Mandarin monolingual infants suggest that even for native learners, tone distinctions are acquired asynchronously. Asynchronies in tone sensitivity have been demonstrated in production (e.g., Wong, 2012a,b, 2013) and in tone discrimination (Tsao, 2017), but not thus far, in infant word learning. Prior studies investigating tone discrimination in Mandarin infants point to emerging asynchronies in tone sensitivity between 6 and 8 and 10 and 12 months of age (Tsao, 2017). Specifically, in Tsao's (2017) tone discrimination study, 10- to 12-month-old Mandarin learning infants were more sensitive to T1 vs. T3 (high, 55, vs. dipping, 214, a different Static-Dynamic contrast to that used in this study) than to the same Dynamic-Dynamic contrast used here (T2 vs. T4; Rising, 35, vs. Falling, 51). Along similar lines, in a familiar word recognition paradigm, Ma et al. (2017) found that Mandarin monolingual toddlers were more sensitive to mispronunciations of familiar words introduced by a Static-Dynamic contrast (T1-T3, 55-214) than by Dynamic-Dynamic contrasts (T3-T4, 214-51 or T2-T3, 35-214). Nevertheless, there is evidence that younger, 6- and 9-month-old, English-language infants discriminate the dynamic-dynamic Thai Rising-Falling tone contrast and do so even better than the static-dynmaic Low-Rising tone contrast (Mattock and Burnham, 2006). This suggests, reminiscent of the Stager and Werker (1997) consonant-based discrimination vs. word learning experiments, that in tone word learning tasks, previously discriminable tone contrasts may be difficuilt to bind to novel words. These findings suggest that, irrespective of tone contrast discriminability, the ease with which Mandarin-learning infants bind tones to novel words is constrained across novel word learning and familiar word recognition, with advantages consistently linked to Static-Dynamic contrasts. Furthermore, our findings suggest that Mandarin monolingual learners orient toward linking native High-Rising tones to words, but not to linking High-Rising Thai tones to novel words. Further research could investigate the question of tone properties more closely by determining whether complex dynamic tones (those involving double dynamic (fall and rise) contours, such as Tone 315 in Thai, are more challenging when associating words with meaning. This possibility is supported by the late encoding of complex Mandarin tones (e.g., 214) even for Mandarin monolingual infants learning novel words (Ma et al., 2017).

Our findings invite the question as to why Mandarin learning infants were insensitive to Rising-Falling contrasts, either in Mandarin or in Thai. One possibility is that this contrast overlaps with the question/statement distinction in Mandarin (Yuan, 2004, 2006; Zeng et al., 2004), which does not differentiate words, but rather specifies communicative intent. It is possible that the structure of the current version of the Switch task (i.e., no contextual cues or other cues to speaker intentionality) renders the rising/falling tone contrast truly ambiguous. If interpreted as a question/statement contrast, language learners should indeed not bind this contrast to word meanings, but if interpreted as a tone contrast, they should rely on it to differentiate words. Prior studies investigating Mandarin learners' abilities to resolve question vs. statement forms with rising and falling tones suggest that their ability to reconcile intonational contrast with lexical tone develops quite late. Only at 4–5 years of age (and not at 3–4 years) do children recognize rising and falling tones regardless of whether they are expressed in rising and falling pitch contours (Singh and Chee, 2016). Even adult speakers of Mandarin demonstrate some processing costs when tone and intonation are potentially confusable (Yuan, 2004). It is therefore possible that Mandarin monolingual infants did not bind rising and falling tone variants to novel words on the grounds that these tones overlap with non-lexical contrasts present in the input. Further research could qualify this possible explanation by testing Mandarin monolingual infants on a non-referential discrimination paradigm to determine whether they could discriminate these tones outside of a word learning context, as per the above mentioned paradigm (Stager and Werker, 1997, Experiment 4). Alternatively, it is possible that language-identifying cues (e.g., carrier sentences in Mandarin) would have facilitated a lexical interpretation of Rising-Falling contrasts.

Indirect support for infants' lexical interpretation of rising-falling contrasts comes from (i) Singh et al. (2016) who, using the same word learning task, found that 18 month monolingual Mandarin learners distinguish both a subtle Dynamic-Dynamic, rising-fall/rise (Mandarin Tone 2, 35 vs. Tone 3, 214), and a Static-Dynamic, high level vs. fall/rise (Tone 1, 55 vs. Tone 3, 214) distinction, and (ii) that when provided with strong referential support and context to signify a lexical tone contrast, 18 month Mandarin learning infants do bind rising and falling tones to word meaning (Singh et al., 2014). Even though non-tone language adults successfully discriminate Rising-Falling lexical tones in Mandarin (T2 vs. T4, Wang et al., 1999), and in Thai (Rising, 315, vs. Falling, 24, Burnham et al., 2014), it is possible that these tones are uniquely complex for infants on account of their substantial overlap with question/statement forms. As suggested by the results here, this intonation-tone overlap may result in greater confusion in infants than adults who may still be engaged in the task of functionally differentiating pitch movements.

### Mandarin-English bilinguals

Findings from bilingual infants suggest a similar advantage for High-Rising contrasts and a similar lack of sensitivity to Rising-Falling contrasts. However, the difference between bilingual and monolingual participants in their perception of the Thai High-Rising contrast suggests important differences in monolingual and bilingual learners' tone percepts. The finding that monolingual Mandarin participants were not sensitive to a Thai tone contrast but bilingual learners were, suggests the possibility of greater phonological flexibility in bilingual infants. This is consistent with previous data suggesting that bilingual infants demonstrate more lenient phonological boundaries for consonant variation (Garcia-Sierra et al., 2011; Petitto et al., 2012; Ferjan Ramírez et al., 2017; Singh, 2017, see also Estes and Hay, 2015 for effects of bilingualism on tone sensitivity). On account of more relaxed phonological boundaries, it is possible that the “grain size” of monolingual tone space may be smaller than that of bilingual infants. Prior studies suggest that the reduced granularity of the bilingual phonological space may facilitate the uptake of words in unfamiliar languages (e.g., Singh, 2017). However, it is possible that this may also complicate language learning. For example, at some point, native and non-native tone variation must be differentiated allowing for the acquisition of more than one tone language. On one hand, it is evident from the current study that native tone sensitivity is *not* reduced in bilingual vs. monolingual learners, so there is no evidence of a bilingual cost to learning Mandarin. On the other hand, it remains to be seen whether a prolonged openness to non-native phonological variation could introduce a cost to learning other tone languages. In other words, the risk-to-opportunity ratio conferred by maintaining phonological flexibility remains to be determined.

### English monolinguals

English monolingual infant learners were impervious to the integration of pitch movements when learning novel words. This is consistent with past studies using the Switch task demonstrating that English monolingual learners bound Mandarin rising and falling contours to word meanings before, but not after 14 months (Hay et al., 2015). However, monolingual English learners, when primed with referential cues, continue to integrate tone into word meaning up to 18 months of age (Singh et al., 2014). Given this, the finding that English learners did not link pitch movements to word meanings is surprising in light of recent studies suggesting that non-tone language (Dutch and English) infants and toddlers become *increasingly* sensitive to a range of Mandarin lexical tone distinctions with age (Chen and Kager, 2015; Liu and Kager, 2015; Chen et al., 2017; Tsao, 2017). However, see also Shi et al. (2017) who found stable discrimination over age of particular Mandarin tone contrasts by French infants. In addition, there is evidence of a decrease in tone sensitivity with age for Thai tones (Mattock and Burnham, 2006) and Cantonese tones (Yeung et al., 2014) in English learning infants.

Thus far, all sources of evidence for increased sensitivity to Mandarin tones in non-tone language learning monolingual infants rests on data from tone discrimination tasks. In this regard, it is of interest here that monolingual English infants *did* respond to pitch differences: they discriminated (albeit between groups) native intonation and non-native tone stimuli as shown by greater attention to (non-native) lexical tone syllables than to (native) intonational syllables. This could be interpreted as evidence that English learning monolingual infants do not treat all sources of pitch variation alike; there may be differences for lexical level (tones) vs. utterance level (intonation). Instead, they may recognize certain pitch contrasts (i.e., lexcial tones) as foreign and unfamiliar leading to a novelty preference for these sources of variation over familiar pitch variation (i.e., intonation). However, the task of interest here was binding differences between lexical tones or intonations to newly learned words, a step beyond discrimination. Thus, it is possible that while non-tone language learning monolingual infants do not consistently demonstrate *perceptual* narrowing for tones (and may show age-related facilitation), they do indeed demonstrate *functional* narrowing for tones such that tone becomes dissociated from word meaning with age. In other words, English learning infants' appreciation of the fact that tone does not serve a lexical function in English may mature in tandem with their increasing sensitivity to pitch movements in non-lexical contexts, such as auditory discrimination. Moreover, the fact that English language non-tone infants did not bind particular intonations to newly learned words in this study may be completely understandable—in English, while intonations are discriminable, they are not used to label words. Pitch discrimination abilities are integral to language comprehension in English (Cutler et al., 1997) and in all languages and as such, infants' selective sensitivity to pitch movements in auditory discrimination tasks but not in lexical tasks may actually reflect maturation and refinement in the functional differentiation of pitch.

## Summary and conclusions

In this set of studies, the range of pitch contrasts to which infants were exposed was broadened from prior studies. The result is a more complex picture than has been revealed by previous research that has focused almost exclusively on the Mandarin rising/falling contour. The results suggest different degrees of tone sensitivity in word learning for different tone contrasts. Findings invite the possibility that tone contrasts that aggregate with intonational contrasts (i.e., rising/falling contrasts) may be more complex to negotiate—particurlarly in the absence of linguistic context—for both native monolingual and bilingual learners alike. In contrast, high and rising tone contrasts were bound to meaning in native tone learners. In comparing infants exposed monolingually and bilingually to Mandarin, our findings point to greater phonological flexibility in tone boundaries by bilingual learners. In sum, our findings extend and expand existing accounts of how infants interpret tone and pitch variation to suggest particularly strong effects of pitch properties on tone sensitivity in novel word learning. These effects appear to be stronger than those of language familiarity in guiding novel word learning.

## Ethics statement

Ethics for testing human participants approved by Western Sydney Human Research Ethics Committee. Approval number—H7330. Infants' caregivers received detailed information about the study and signed an informed consent form. Infants were accompanied by their caregivers at all times during their visit to the four labs (U. Lancaster, NUS, Sunway U, and Western Sydney U) and the task was discontinued immediately if the infants showed any signs of discomfort or their caregivers wished so.

## Author contributions

DB: leader of the project; lead chief investigator on funded grant that provided funds for the project; with KM, designed the experiments and stimuli; management of all aspects of the project including experiment running experiment, analyzing results, and writing the manuscript. LS: supervision of collection of bilingual data and monolingual Mandarin data in Singapore; management of stimulus material collection in Singapore; significant writing of manuscript, especially the Introduction; intellectual input to the project, especially to the interpretation of the results. KM: chief investigator on funded grant that provided funds for the project; with DB, designed the experiments and stimuli; management of monolingual English data collection at Lancaster University; editing and commenting on manuscript. PW: significant input to the design of the experiments; supervision of collection of bilingual data and monolingual Mandarin data in Malaysia; management of stimulus material collection in Malaysia; editing and commenting on manuscript. MK: data collection of monolingual English data at Lancaster University; supervision of monolingual English data collection at MARCS Institute, WSU; co-ordination of bilingual data collection between Malaysia and Singapore; analysis of data; preparation of figures; editing and commenting on manuscript.

### Conflict of interest statement

The authors declare that the research was conducted in the absence of any commercial or financial relationships that could be construed as a potential conflict of interest.

## Footnotes

^1^To check this, we conducted a 2 × 2 × 2 ANCOVA with native/non-native, Static-Dynamic/Dynamic-Dynamic as the between-subjects variables, Same/Switch as the within-subjects variable, and percentage of Mandarin exposure as the covariate. The results were identical to the reported results. There was no main effect of Same/Switch, *F*_(1, 27)_ = 0.473, *p* = 0.497, η^2^ = 0.017, native/non-native, *F*_(1, 27)_ = 1.688, *p* = 0.25, *n* = 0.059, tone type, *F*_(1, 27)_ = 0.195, *p* = .662, η^2^ = 0.007, or exposure to Mandarin, *F*_(1, 27)_ = 0.128, *p* = 0.724, η^2^ = 0.005, and no Same/Switch by native/non-native, *F*_(1, 27)_ = 2.207, *p* = 0.080, η^2^ = 0.109, Same/Switch by exposure to Mandarin, *F*_(1, 27)_ = 0.240, *p* = 0.628, η^2^ = 0.009, native/non-native by tone type, *F*_(1, 27)_ = 0.702, *p* = 0.410, η^2^ = 0.025, or Same/Switch by native/non-native by tone type, *F*_(1, 27)_ = 0.038, *p* = 0.847, η^2^ = 0.001, interactions. The only significant interaction was Same/Switch by Tone Type, *F*_(1, 27)_ = 9.710, *p* = 0.004, η^2^ = 0.265, as reported.
